# Recovery of infectious virus from full-length cowpox virus (CPXV) DNA cloned as a bacterial artificial chromosome (BAC)

**DOI:** 10.1186/1297-9716-42-3

**Published:** 2011-01-11

**Authors:** Swaantje J Roth, Dirk Höper, Martin Beer, Silke Feineis, B Karsten Tischer, Nikolaus Osterrieder

**Affiliations:** 1 Institut für Virologie, Freie Universität Berlin; Philippstrasse 13, Haus 18; 10115 Berlin, Germany; 2 Institut für Virusdiagnostik, Friedrich-Loeffler-Institut, Südufer 10, 17493 Greifswald-Insel Riems, Germany

## Abstract

Transmission from pet rats and cats to humans as well as severe infection in felids and other animal species have recently drawn increasing attention to cowpox virus (CPXV). We report the cloning of the entire genome of cowpox virus strain Brighton Red (BR) as a bacterial artificial chromosome (BAC) in *Escherichia coli *and the recovery of infectious virus from cloned DNA. Generation of a full-length CPXV DNA clone was achieved by first introducing a mini-F vector, which allows maintenance of large circular DNA in *E. coli*, into the thymidine kinase locus of CPXV by homologous recombination. Circular replication intermediates were then electroporated into *E. coli *DH10B cells. Upon successful establishment of the infectious BR clone, we modified the full-length clone such that recombination-mediated excision of bacterial sequences can occur upon transfection in eukaryotic cells. This self-excision of the bacterial replicon is made possible by a sequence duplication within mini-F sequences and allows recovery of recombinant virus progeny without remaining marker or vector sequences. The in vitro growth properties of viruses derived from both BAC clones were determined and found to be virtually indistinguishable from those of parental, wild-type BR. Finally, the complete genomic sequence of the infectious clone was determined and the cloned viral genome was shown to be identical to that of the parental virus. In summary, the generated infectious clone will greatly facilitate studies on individual genes and pathogenesis of CPXV. Moreover, the vector potential of CPXV can now be more systematically explored using this newly generated tool.

## Introduction

Cowpox virus (CPXV) belongs to the family *Poxviridae*, a family of large double- stranded DNA viruses replicating in the cytoplasm. It is endemic in Eurasia and reportedly transmitted from rodents as the reservoir host to other mammals. CPXV causes disease in felids, zoo animals, man and, despite its name, only very rarely in cattle. Not only recent outbreaks in humans but also its close relationship to other important members of the genus such as variola virus have drawn attention to this virus as a model, especially regarding its impressive abilities to evade host immunity [1-3].

Bacterial artificial chromosomes (BAC), first published by Shizuya et al. in 1992 [4], allow the stable maintenance of large plasmids of up to 300 kbp in size at low copy numbers in *Escherichia coli*. The genome of a herpesvirus, human cytomegalovirus (HCMV), was the first full-length viral genome cloned as a BAC in 1997 [5]. Infectious genomes cloned as BAC have proven a useful tool in molecular virology, both for investigations into viral pathogenesis as well as for applications in vaccination and gene therapy, because mutations can be introduced with ease using recombination or transposon systems of *E. coli *[6].

With respect to poxviruses, the cloning and successful recovery of infectious BAC was published for vaccinia virus strains Western Reserve [7,8] and for modified vaccinia virus Ankara (MVA), an attenuated vaccinia virus strain that is replication-deficient in most cell types [9]. Here, we show the generation and characterization of the first infectious clone of a cowpox virus that contains the complete genome of CPXV strain Brighton Red (BR). The BAC allows the application of bacterial mutagenesis procedures including minimal modifications and markerless approaches. With the help of the transcriptional machinery of fowlpox virus (FWPV), infectious CPXV could be recovered in cell culture. Since it is desirable to avoid any foreign sequences in reconstituted viruses, we modified the parental CPXV BAC into a self-excisable construct by introducing a duplication of flanking sequences in inverse orientation into the mini-F cassette, an approach that was previously shown for infectious herpesvirus and poxvirus genomes [10]. Self-excision of mini-F sequences was efficient upon reconstitution in eukaryotic cells and virus progeny derived from cloned genomes therefore is genetically and phenotypically indistinguishable from parental CPXV BR.

## Materials and methods

### Cell lines and viruses

African green monkey Vero 76 cells (Collection of Cell Lines in Veterinary Medicine, Friedrich-Loeffler Institute, Greifswald-Insel Riems, Germany) were kept in Eagle's minimal essential medium (MEM, Biochrom, Berlin, Germany) supplemented with 5-10% fetal bovine serum (FBS, Biochrom). Primary chicken embryo cells (CEC) were prepared from 11-day-old embryos according to standard procedures and cultured in MEM containing 10% FBS [11].

CPXV strain Brighton Red (AF428758), kindly provided by Dr Philippa Beard, University of Edinburgh, UK, was propagated on Vero cells and FWPV (Nobilis-PD, strain WP, Intervet, Boxmeer, The Netherlands; kindly provided by Dr D. Lüschow, Freie Universität Berlin, Germany), on CEC.

### Bacteria and plasmid constructs

#### Plasmids for generation of the CPXV BAC pBRf

The pBeloBAC11-derived mini-F construct was kindly provided by Dr M. Cottingham, Jenner Institute Oxford, UK. For construction of the transfer plasmid used for the generation of recombinant thymidine kinase (TK)-negative CPXV (BR.TK-), the nonessential TK gene (*BR 106*) was amplified with primers #46 and #47 (Table 1) and initially cloned into pCRII (Invitrogen, Darmstadt, Germany) (plasmid #5). An inverse PCR, using primers oligonucleotides #48 and #49, led to the introduction of an *Fse*I restriction site located centrally within the TK gene after religation (plasmid #6). An *Fse*I restriction fragment of the pBeloBAC11 derivate, containing the mini-F cassette with the chloramphenicol resistence gene (*cat*), *loxP *sites and the green fluorescent protein gene (*gfp*) under the control of the late FWPV promoter p4B [9], was ligated into the single *Fse*I restriction site of plasmid #6. Colonies containing the resulting, complete transfer plasmid (plasmid #7) were selected for their triple resistance to ampicillin, kanamycin and chloramphenicol.

**Table 1:** Primers used in this study

Oligo #	Sequence 5'-3'	Comments
oligo #046 forward	TCCTTAATTAAGACAACTCAAACATCTGCGTTATC	
oligo #047 reverse	TCCTTAATTAAGCAGTACTAGGTTCATTTCCTCCT	
oligo #048 forward	TTTAAAGGCCGGCCACAGTTCTTTCCAGACATTGTTGA	*Fse*I site underlined
oligo #049 reverse	TTTAAAGGCCGGCCTTCATCGATACCTATCACGG	*Fse*I site underlined
oligo #237 forward	GAGCCCACGTTTAAACATTCTT	
oligo #238 reverse	GGTAACATAATACCCTTTTCCTGAA	mini-F
oligo #239 forward	TCGTATGTTGTGTGGAATTGTGA	mini-F
oligo #240 reverse	ACCGTGTGGAATATAAAAGTCCT	
oligo #264 reverse	CGATAATAGATACGGAACGGGAC	TK
oligo #265 forward	TTACGTTGAAATGTCCCATCAA	TK

#### Plasmids for generation of self-excisable CPXV BAC pBR

The transfer plasmid for introduction of an inverse duplication of the TK flanks into the mini-F sequence present in full-length CPXV.BR BAC (pBRf) was based on plasmid pEP*MCS*-in-Belo as described previously [10]. The transfer construct contains mini-F-derived sequence flanks A and B of approximately 830 bp in length as well as the kanamycin resistance gene *aphAI *with an adjoining *I-Sce*I restriction site. The *aphAI-I-Sce*I cassette is flanked by duplication sequences utilized for *E. coli *recombineering, *I-Ceu*I restriction sites frame the complete construct. The TK fragment was cut out of plasmid #5 using *BamH*I and *Pst*I restriction sites and ligated into the multiple cloning site of pEP*MCS*-in-Belo (plasmid #10). The duplication was inserted into the mini-F cassette in inverse orientation relative to original viral sequences in pBRf.

#### Bacteria and transformation

Regular plasmids were grown and maintained in *E. coli *Top10 cells (Invitrogen). For generation of an infectious CPXV full length clone, pBRf, electrocompetent *E. coli *DH10B cells (MegaX, Invitrogen) were electroporated with viral DNA at 1300 V and 100 Ω resistance (Biorad, München, Germany). For generation of pBR, two-step Red recombination was applied after transfer of pBR into *E. coli *GS1783 cells [12]. All recombination procedures for two-step *en passant *recombination were performed exactly as described previously [13].

### Preparation of viral and plasmid DNA, restriction fragment length polymorphism (RFLP) analysis and Southern blotting

Viral DNA for electroporation and generation of pBRf was isolated from cells infected with BR.TK- after addition of 100 μM IβT (isatin-β-thiosemicarbazone, Chemos GmbH, Regenstauf, Germany) at 3 h post infection (pi). Cells were harvested by scraping at 48 h pi and the cell pellet was incubated in lysis buffer (0.02 M Tris HCl (pH 8.0); 0.01 M EDTA; 0.75% SDS; 0.65 mg/mL proteinase K) at 55°C for 5 h. Nucleic acids were extracted by addition of supersaturated NaCl to a final concentration of 2 M and precipitated with isopropanol (final concentration: 50%). The same procedure without addition of IβT was followed for the extraction of viral DNA for PCR analysis and determination of restriction fragment length polymorphisms (RFLP).

Plasmid or BAC DNA for cloning and standard applications such as restriction enzyme digests was extracted by the alkaline lysis method, variably including or omitting phase separation and removal of cellular debris with phenol-chloroform [14]. For whole genome sequencing, large scale BAC DNA purifications were carried out using the plasmid midi kit (Qiagen, Hilden, Germany) following the manufacturer's instructions. For determination of RFLP, DNA cut with various restriction enzymes was separated by electrophoresis for 14 h at 35 V in 0.8% agarose gels using 1× TAE buffer (40 mM Tris; 20 mM acetic acid; 1 mM EDTA; 50× TAE sock solution pH 8.4).

Southern blotting with a digoxigenin (DIG) labeled TK PCR probe was done using the DIG PCR Labeling Kit and the DIG Nucleic Acid Detection Kit (Roche, Mannheim, Germany) exactly following the manufacturer's instructions.

### Reconstitution of infectious virus

Transfections for the generation of recombinant virus as well as for recovery of virus from BAC DNA were carried out with approximately 2 μg of purified plasmid or BAC DNA and 4 μL FuGene (Roche) according to the manufacturer's instructions. For virus reconstitution, 1 × 10^6 ^Vero cells seeded in one well of a 6-well plate were infected with 100-1 000 plaque-forming units (pfu) of FWPV at 1 to 5 h before or after transfection.

### Pyrosequencing

A DNA library for sequencing with Genome Sequencer FLX (Roche) was prepared from enriched and purified BAC DNA according to an established protocol [15]. The resulting dsDNA library was then bound to library capture beads and, subsequently, a single-stranded template DNA (ssDNA) library was recovered. The ssDNA library was prepared for sequencing and sequenced according to the manufacturer's instructions. The obtained raw sequence data were assembled using the GS assembler software Newbler (v. 2.0.00.22; Roche). Assembled contigs were further analyzed with VectorNTI Advance 11 (Invitrogen).

### Characterization of virus growth properties

Single- and multi-step growth kinetics of reconstituted viruses were carried out on Vero cells using multiplicities of infection (MOI) of 3 or 0.01, respectively. Cells were infected, washed three times with PBS 90 min after infection, and supplied with 1 mL of fresh cell culture medium. The 0 h time point was taken immediately after addition of new medium. Cell culture supernatant was harvested, subjected to low-speed centrifugation (8 min, 6 000 × *g*) to remove cells and cellular debris, and 1 mL of fresh medium per well were added. Both the cellular fraction and cleared supernatants were treated by freezing (-70°C) and thawing (37°C) three times before centrifugation and followed by quantification in plaque assays.

End point titers were determined in a similar manner. Virus was added to cells at an MOI of 3, 0.1 or 0.01, and cells were harvested at 24, 48 and 72 h after the washing procedure that was done 90 min after addition of virus as described above.

For plaque size determinations, 1 × 10^6 ^Vero cells were infected with 100 pfu per well of a 6-well plate and overlaid 90 min pi with carboxymethyl cellulose (CMC, final concentration: 0.8% in MEM with 3.5% FBS). Plaque images were taken at 48 h pi using the Zeiss Axiovert 25 (Carl Zeiss, Jena, Germany) equipped with a CCD camera (AxioCam MRm, Zeiss), processed with the Axiovision software (Zeiss), and analyzed using ImageJ software http://rsb.info.nih.gov/ij/. Statistical analyses of virus growth kinetics and plaque areas were done using the Student's *t*-test http://www.graphpad.com.

## Results

### Generation of recombinant CPXV BR.TK- harboring mini-F vector sequences

**Figure 1:**
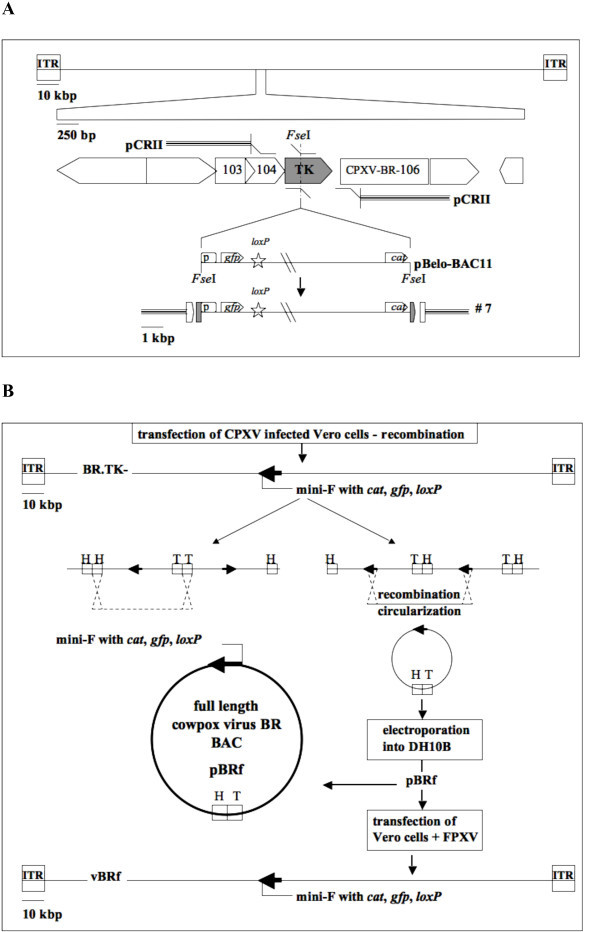
**Schematic presentation of rationale for cloning of cowpox virus infectious clone**. A. Shown is a schematic of the full-length CPXV.BR genome and the TK locus in greater detail. In a first step, the viral TK was amplified by PCR, cloned into the pCRII vector, which was then used for an inverse PCR to introduce an *Fse*I restriction site into TK. Mini-F vector sequences containing *gfp *under the control of the late 4B promoter were finally inserted into the singular *Fse*I site to obtain transfer plasmid #7. B. Shown are the constructs and the strategy used to obtain recombinant virus BR.TK- and the full-length CPXV.BR BAC pBRf. Recombinant BR.TK- was used to infect Vero cells. Infection resulted in the formation of replication intermediates, concatemers, some of which were circularized. Viral DNA extracted from cells infected with BR.TK- was electroporated into *E. coli *DH10B giving rise to pBRf. The full-length CPXV BAC clone was ultimately transfected into Vero cells using FWPV as a helper. Scale bars indicate the sizes of the molecules. Abbreviations: ITR, inverted terminal repeats; *cat*, chloramphenicol resistance gene; *gfp*, green fluorescent protein gene: *loxP*, loxP sites; H, "head", T, "tail" orientation of the ITR present in the replicative intermediates.

Vero cells infected with CPXV strain BR were transfected with transfer plasmid #7 (Figure 1A) at 3 h pi. Plaque picking and passaging of end-point dilutions of recombinant progeny virus identified by GFP expression were carried out in an alternating fashion. Pure recombinant BR.TK-, which carries mini-F sequences from modified pBeloBAC11 inserted within TK-encoding sequences, was obtained after 8 cycles of plaque and end-point purification (Figure 1B).

### Generation of an infectious full-length clone of CPXV BR (pBRf) and reconstitution of recombinant virus (vBRf) from cloned DNA

**Figure 2:**
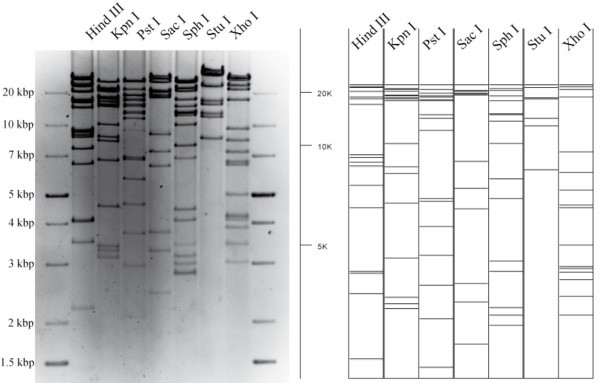
**Restriction fragment analysis of full-length BAC clone pBRf**. A. BAC DNA was isolated and digested with seven different enzymes (HindIII, KpnI, PstI, SacI, SphI, StuI, XhoI) and separated on a 0.8% agarose gel. Sizes of a molecular weight marker (generuler 1-kb plus DNA ladder, Fermentas) are given. B. Patterns corresponded exactly to the predictions based on the complete CPXV.BR genome (#NC_003663) as shown by *in silico *digests using VectorNTI.

The sequence of manipulations resulting in the generation of a full-length CPXV infectious clone, including the cloning strategy and the mini-F vector recombination into the viral genome, are illustrated in Figure 1. Vero cells were infected with recombinant virus BR.TK- and supplemented with IβT in order to accumulate unresolved concatemeric replication intermediates through the interference of the drug with late transcription [16,17] (Figure 1B). Among the accumulated concatemers, the small fraction of desired head-to-tail concatemers was increased accordingly. Only head-to-tail concatemers can harbor complete CPXV genomes and the mini-F sequence in the same orientation. Consequently, the likelihood of homologous recombination of sequences with identical orientation within head-to-tail concatemeric DNA is higher and can lead to circularization of the full-length genome. Viral DNA was electroporated into electrocompetent *E. coli *DH10B cells and the full-length CPXV BAC, pBRf, was obtained (Figure 1B). Restriction enzyme digestion and RFLP analysis using 7 different enzymes proved establishment and faithful maintenance of the complete CPXV genome as a low-copy BAC in *E. coli*. The RFLP's obtained for pBRf after digestion and 0.8% agarose gel electrophoresis using HindIII, KpnI, PstI, SacI, SphI, StuI and XhoI exactly matched those from in silico predictions (Figure 2).

After confirming the integrity of the full-length pBRf clone, virus was successfully reconstituted from DNA using FWPV as helper virus. BAC DNA was transfected into 1 × 10^6 ^Vero cells that were infected with 100-1 000 PFU of FWPV several hours before or after infection. Formation of GFP-expressing virus plaques showed that virus derived from pBRf, termed vBRf, was fully replication-competent. Integrity of the complete vBRf genome including the mini-F sequences was proven by demonstration of the expected restriction fragment patterns and Southern blot analysis (see below). We therefore concluded that the complete CPXV.BR genome was successfully established in *E. coli *and that fully replication-competent CPXV was reconstituted from viral DNA cloned as a BAC.

### Construction of self-excisable CPXV BAC pBR and generation of recombinant vBR virus

**Figure 3:**
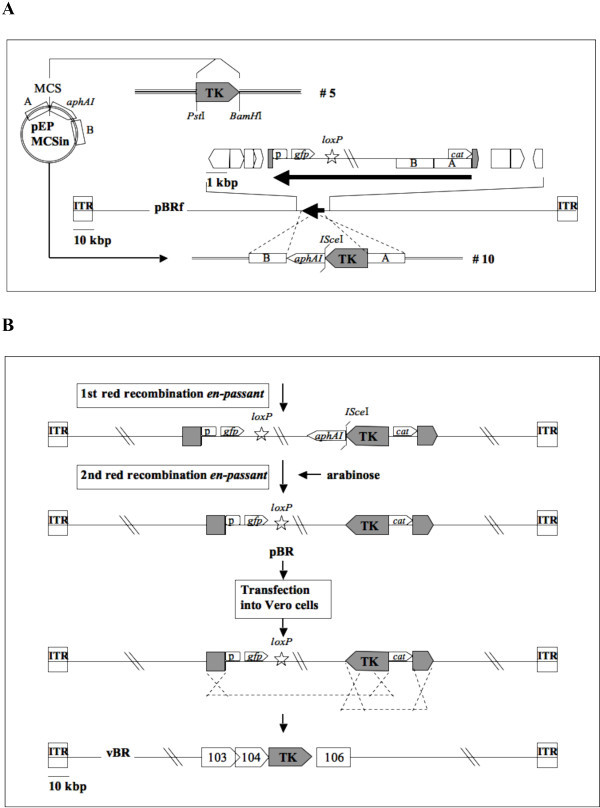
**Schematic of the modification of pBRf to arrive at a self-excisable construct (pBR)**. A. The TK sequences present in plasmid #5 were inserted into plasmid pEP*MCS*-in-Belo containing flanks A and B homologous to sequences within in the mini-F vector present in pBRf as well as an *I-Sce*I site and *aphAI*, which confers resistance to kanamycin, resulting in plasmid #10. B. The construct was then integrated into the mini-F sequence of pBRf in inverse orientation via *en passant *mutagenesis. After transfection of the generated construct in eukaryotic cells, vector sequences are lost through two antiparallel recombination events, which are indicated by the cross-over lines.

**Figure 4:**
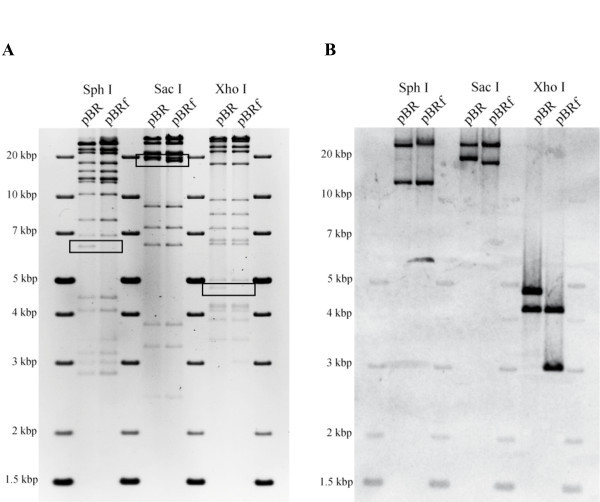
**Restriction fragment analysis of of pBR and pBRf**. A. BAC DNA was digested with SphI, SacI or XhoI and fragments separated by 0.8% agarose gel electrophoresis. Changes of the restriction pattern due to the inserted TK duplication are marked with a box. Sizes of a molecular weight marker (generuler 1-kb plus DNA ladder, Fermentas) are given. B. Southern blot analysis after transfer of the gel shown in (A) and using a TK DIG labeled probe. The expected sizes of the fragments were identified (see text). Sizes of a molecular weight marker (generuler 1-kb plus DNA ladder, Fermentas) are given.

In order to construct a CPXV BAC that would allow self-excision of the mini-F vector sequences upon virus reconstitution in eukaryotic cells, the full-length pBRf BAC was transferred into recombination-competent *E. coli *GS1783 cells [12]. Using markerless two-step Red recombination [13], an inverse duplication of TK sequences identical to the viral sequence flanks on either side of the inserted mini-F sequences present in pBRf was introduced within the mini-F cassette itself. To achieve this introduction, transfer plasmid pEPMCS_TK (plasmid #10) was digested with I-CeuI and the resulting fragment (Figure 3A) was electroporated into recombination competent GS1783 harboring pBRf. In a first Red recombination, the TK sequences, together with *aphAI *and an *I-Sce*I site immediately adjoining the resistance gene and short flanking sequences, were introduced into the mini-F sequences of pBRf. Recombination occurred between homologous sequences present within the mini-F (Figure 3B). Resulting colonies were screened for resistance against kanamycin and chloramphenicol and BAC DNA from double-resistant colonies was extracted and examined by RFLP. Colonies with BAC RFLP patterns matching those for the predicted cointegrates were resolved, taking advantage of the homing endonuclease *I-Sce*I site located upstream of the *aphAI*. Cleavage at the site induces a double strand break resulting in excision of the resistance gene by homologous recombination between duplication of sequences up- and downstream of the *aphAI-I-SceI *casette in a second recombination event [13]. After induction of I-SceI cleavage, resulting colonies were screened for the loss of kanamycin resistance and the final construct, termed pBR, was analyzed by RFLP and compared to the restriction enzyme pattern of the parental pBRf BAC. Comparison of restriction enzyme profiles obtained for SphI, SacI and XhoI confirmed successful integration of the duplicated sequences within the mini-F vector sequences (Figure 4). Insertion of the duplication of TK sequences led to an additional 6.3 kbp band, enlargement of a 17.9 kbp to a 19.6 kbp band, and the appearance of a 4.7 kbp band, respectively (Figure 4). Successful insertion was also proven by Southern blot analysis using a TK probe, which demonstrated the presence of the additional TK sequences. In the SphI digest, the 37.8 kbp band in pBRf is reduced in size to a 33.2 kbp band, whilst the 11.9 kbp band is consistently present in both pBRf and pBR as predicted (Figure 4). In the case of SacI digested DNA, the 36.5 kbp band present in pBRf is also present in pBR, whereas the 17.9 kbp band is enlarged and 19.6 kbp in size in pBR (Figure 4). A very clear difference is seen in the XhoI patterns of pBRf and pBR, where a 3 kbp band increases in size to 4.7 kbp following insertion of the TK duplication (Figure 4). From the results we concluded that the TK duplication was correctly inserted into the pBRf, which was further verified by DNA sequencing of the modified mini-F vector sequences in pBR (data not shown).

**Figure 5:**
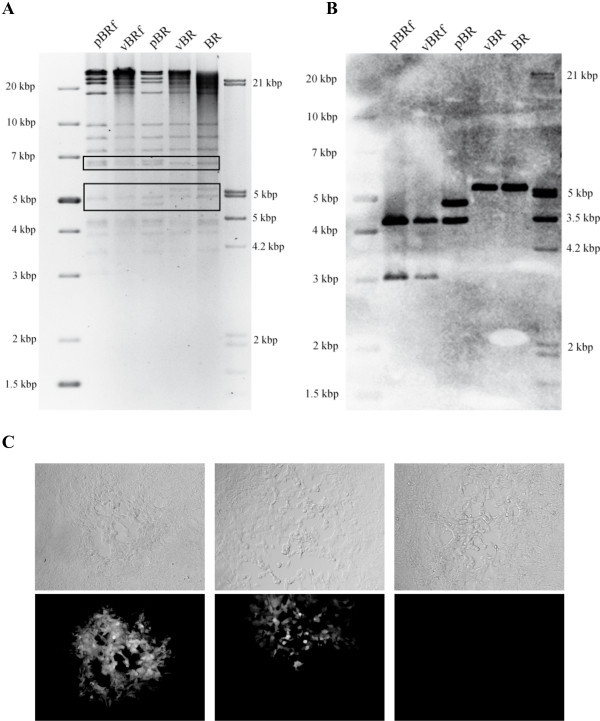
**RFLP analysis of pBR- and pBRf-derived viruses and characterization of mini-F loss in pBR-derived viruses**. A. BAC DNA or viral DNA prepared from infected Vero cells was digested with XhoI and separated by 0.8% agarose gel electrophoresis. The lanes contain the DNA's from BAC DNA (pBRf, pBR), viruses reconstituted from cloned DNA (vBRf, vBR) or parental CPXV.BR DNA (BR). Changes of the restriction pattern due to the presence and therefore disruption of TK of absence of mini-F sequences are marked with boxes (see text). B. Southern blot analysis after transfer of the gel shown in (A) and using a DIG labeled TK probe. The changes seen with a DIG labeled TK probe show the expected changes due to the disruption of TK with mini-F sequences (pBRf, vBRf), the introduction of an inverse TK sequence (pBR), and the loss of any bacterial sequences in vBR (see text). Sizes of a molecular weight marker (generuler 1-kb plus DNA ladder, Fermentas) are given. C. Development of GFP fluorescence in plaques following removal of mini-F sequences. Initially, all plaques exhibit GFP fluorescence (plaque in left panels), which is then partially lost (plaque in middle panels) or completely absent (plaque in right panels). Identical plaques were photographed under fluorescent light (upper panels) or using phase contrast (lower panels) with a Zeiss Axiovert microscope and a CCD camera (Zeiss).

**Figure 6:**
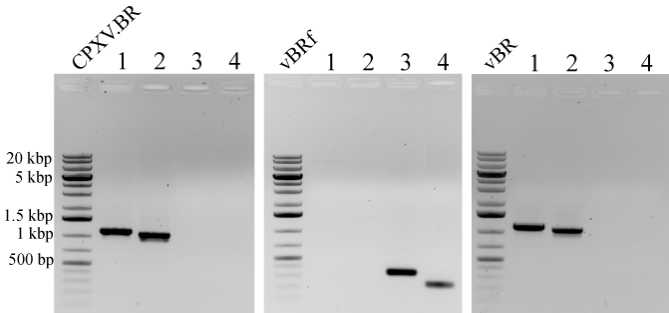
**PCR analysis for verification of presence of mini-F sequences in CPXV.BR, vBRf or vBR**. DNA isolated from cells infected with CPXV.BR, vBRf or vBR was subjected to PCR targeting TK and the mini-F vector. Lanes 1 and 2 contain PCR amplicons using primer pairs #237+#265 or #264+#240, respectively, which target full-length TK. Bands at sizes of 1,126 bp or 1,054 bp, respectively, are obtained in the case that mini-F sequences are absent in the DNA analyzed. Lanes 3 and 4 using primer pairs #264+#238 or #239+#265) only yield a PCR product in the case that the mini-F sequence is still present and result in bands of 324 bp and 210 bp, respectively. Gel images for the individual virus DNAs are shown and the size of a molecular weight marker (generuler 1-kb plus DNA ladder, Fermentas) are given.

Vero cells were transfected with pBR, and upon reconstitution using FWPV as a helper, non-fluorescent plaques, indicative of the loss of the mini-F plasmid as GFP expression was absent, were already observed in the first passage after transfection (Figure 5). After three passages, a homogenous virus population was obtained in which none of the virus plaques examined showed GFP fluorescence. Virus progeny from those plaques was thus suspected to have lost mini-F sequences through a recombination event mediated by the inverse TK duplication. Bacterial and virus DNA was therefore digested with XhoI (Figure 5) or KpnI (data not shown) and RFLP and Southern blotting were performed. In both RFLP and Southern blot analyses, DNA obtained from vBR-infected cells and cells infected with parental wild-type BR showed the same pattern, which indicated the overall similarity of nucleotide sequences of virus obtained from cloned DNA and the original CPXV. Absence of residual vector sequences was finally confirmed by PCR targeting the mini-F and TK loci with primer pairs either targeting TK sequences or both mini-F and TK sequences. Presence of mini-F sequences was readily detectable by PCR in vBRf obtained after reconstitution from pBRf (Figure 6). In contrast, DNA obtained from cells infected with recombinant vBR virus or wild-type virus BR lacked the corresponding bands and instead specified bands following PCR amplification targeting sequences at both the extreme 5' and 3' end of the TK gene (Figure 6). From the results of the RFLP patterns and the PCR we concluded that mini-F sequences were indeed completely absent in virus progeny reconstituted from pBR and was therefore indistinguishable from parental CPXV.BR.

### Nucleotide sequence of pBR

Results from RFLP's and the following Southern blot analysis were consistent with the predicted restriction enzyme fragment patterns, and sizes of restriction fragments of pBRf, vBRf, pBR and vBR were identical to those present in CPXV.BR or were fully consistent with the introduced sequences (pBRf, vBRf, pBR, Figures. 2, 4 and 5). To further evaluate the integrity of the generated infectious clones, whole genome sequencing of pBR and comparison with the published CPXV.BR sequence (accession #NC_003663) was performed. pBR DNA was purified and subjected to pyrosequencing. Assembly of the contigs revealed only very few mismatches and changes when compared to the published sequences. We were unable to sequence approximately 2.5 kb on either end of the pBR genome that contain highly repetitive, noncoding sequences needed for viral DNA amplification during the virus replication cycle [18]. The sequences are known to be highly variable, but were shown to be functional as proven by the fact that we were able to reconstitute virus *de novo *from the infectious clone. We then targeted the differences between the determined pBR and published CPXV.BR sequence as well as regions in the nucleotide sequence that had remained ambiguous after pyrosequencing. Regions with unresolved ambiguities were amplified by PCR and sequenced by standard chain termination protocols according to Sanger (StarSeq, Mainz, Germany). We discovered that sequence changes were caused by pyrosequencing errors in the majority of the cases. However, we identified a total of 7 remaining non-silent sequence variations in pBRf when compared to the published sequence (Table 2). Those regions were then analyzed by PCR amplification and sequencing of the corresponding regions of parental DNA derived from CPXV.BR used for the generation of the BAC. Comparison of the aligned sequences of CPXV.BR and pBR revealed that the sequences of the infectious BAC clone did not differ from that of the original virus but only from the Genbank reference sequence (Table 2) with the exception of two silent nucleotide changes in the TK locus that were introduced by the initial PCR for cloning of the flanking regions for introduction of the mini-F vector. As codon usage was not affected by the two nucleotide exchanges and as the nucleotide substitutions can possibly be used for genetic identification of BAC-derived viruses, we refrained from repairing the PCR errors. We find it remarkable that only a total of 7 nucleotide differences between the pBR and the published CPXV.BR were identified and are unable to determine the reason for the changes at the present time. However, it is likely that passaging of virus lead to changes in the indicated genes, particularly as affected genes are exclusively genes involved in immunomodulation and in vivo host range determination for which evolutionary pressure is absent under the condition of propagation in cultured cells [19].

**Table 2:** List of nucleotide sequence differences of CPXV.BR and cloned pBR when compared to the published BR sequence.

**A. **Changes in coding sequences				
**Gene (according to NC_003663)**	**Function**	**Modification (nt position)**	**Present in parental virus**	**Effect**

006	ankyrin^a^	C to A (5 038)	no	silent
019	ankyrin	C to T (21 015)	yes	Glu to Lys, not in ankyrin repeats
035	kelch-like	ins. T (36 656)	yes	split protein, resembles situation in other orthopox viruses
079	glutaredoxin (GRX) family	C to T (76 314)	yes	Asp to Asn, polymorphic region of the protein
089	protein G1 (Peptidase)	T to C (87 650)	yes	Asn to Asp, like in 91-3
105	tk	T to C (97 522)	no	silent, PCR error
106	polyA polymerase small SU	(105 299)	no	silent, PCR error
139	poxvirus early transcription factor (VETF)	Insertion : T (142 924)	yes	C-terminal amino acid sequence changed from RAQIN to KSTNKLNN
193	kelch-like	C to T (189 440)	yes	Ser to Phe, polymorphic region of the protein
215	kelch-like	C to T (213 275)	yes	Pro to Ser, polymorphic region of the protein
225	ankyrin^a^	G to T (229 788)	no	silent

B. Changes in non-coding sequences				

Modification (nt position)		Comment		

Insertion : TG (8 899-8 900)^b^		poly-TG repeat, polymorphic in viral DNA		
C to T (58 974)				
Insertion : T (65 922)		poly-T stretch		
Insertion : CA (225 926-225 927)^b^		poly-CA repeat, polymorphic in viral DNA		
Insertion : TG (8 899-8 900)^b^		poly-TG repeat, polymorphic in viral DNA		

### Phenotypic characterization of recombinant CPXV.BR derived from pBRf or pBR

**Figure 7:**
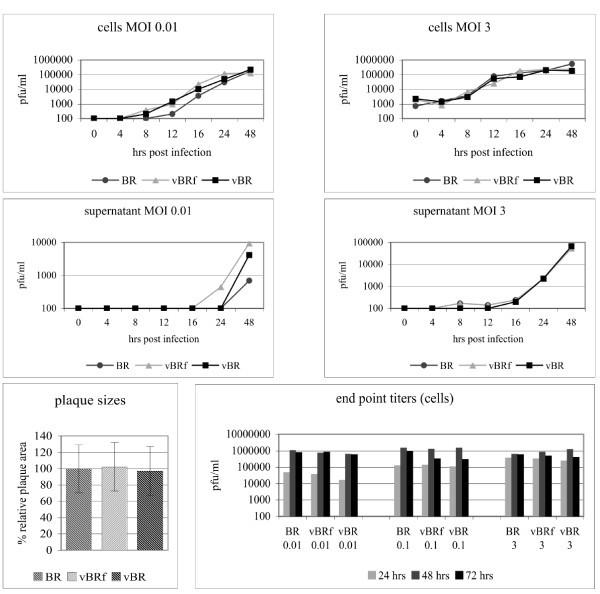
**Characterization of growth properties of vBRf, vBR in comparison to wild-type virus CPXV.BR**. A. Vero cells were infected at the indicated MOI (0.01 or 3) with the various viruses, and virus titers were determined from culture supernatants or the infected-cell pellets at the time points given. Shown are means of two independent experiments and the standard deviations (error bars). B. Plaque areas were determined by measuring a total of 40 plaques induced by each virus at 72 h pi. Plaques were stained with crystal violet and plaque images were taken using the Axiovert 25 microscope and CCD camera (Zeiss). Plaque sizes were measured using ImageJ. Mean plaque sizes induced by CPXV.BR were calculated and set to a 100%. Plaque areas determined for vBRf or vBR are shown relative to the CPXV.BR value. Shown are means and standard deviations (error bars) for each virus. C. End point titers were determined after infection of Vero cells with the indicated viruses at an MOI of 0.01, 0.1 or 3. Virus titers were determined from whole cell lysates at 24, 48 or 72 h pi.

In the final set of experiments, we determined the growth characteristics of the viruses generated from infectious clones in comparison with parental CPXV.BR. By and large, the in vitro characteristics of viruses reconstituted from infectious did not differ significantly from those of CPXV.BR. Single-step growth kinetics performed by infecting Vero cells at an MOI of 3 revealed that both BAC-derived viruses, vBRf and vBR, reached extracellular virus titers between 1 000 and 100 000 pfu/mL and cell-associated virus titers between 100 000 and 1 000 000 pfu/mL at 48 h pi. Titers at all time points after infection were virtually indistinguishable from those of parental CPXV.BR (Figure 7). Similarly, multi-step growth kinetics performed by infection of Vero cells at an MOI of 0.1 with the various viruses did not reveal significant differences between the viruses either, and titers recorded for vBRf, vBR and CPXV.BR were highly similar in the case of both extracellular and cell-associated virus titers (Figure 7). End-point titer determinations at 24 h, 48 h and 72 h pi obtained after infection at MOI of 0.01, 0.1 and 3 showed highest titers when we infected at an MOI of 0.1 and again proved that the growth characteristics of vBRf and vBR were virtually indistinguishable from those of wild-type virus CPXV.BR. Finally, the plaque morphology and plaque areas determined for the three viruses were highly similar and not significantly different from each other (vBRf vs. CPXV.BR: p = 0.7409, vBR vs. CPXV.BR: p = 0.6688, n = 40; Figure 7). From the data of the virus growth kinetics and plaque area comparisons we concluded that manipulation of CPXV.BR by insertion of the mini-F sequences and/or deletion of the TK gene did not have a measurable effect on virus replication in Vero cells. Hence, the TK gene of CPXV.BR is completely dispensable for growth in cultured Vero cells and CPXV reconstituted from cloned BAC DNA behaves virtually identical to its parental wild-type virus in the culture system examined.

## Discussion

We here describe the first BAC clone containing the complete genomic sequence of CPXV.BR. Furthermore we report an infectious clone in which bacterial sequences are lost via homologous recombination events in eukaryotic cells and therefore present a BAC that, upon reconstitution in cell culture, does not contain any vector or marker sequences and therefore is genetically indistinguishable from the parental virus.

For generation of the infectious clone, the thymidine kinase locus was chosen based on data from other orthopox viruses, which had shown the nonessential nature of the gene when virus is propagated in cultured cells [20]. Our results on the generation of a TK-negative CPXV.BR virus, achieved through the insertion of bacterial vector sequences into the TK locus, lend support to the non-essential function of this gene in CPXV. Furthermore, detailed analysis of the growth characteristics showed that even vBRf, in which the TK is disrupted by the 9 kbp insertion of the mini-F vector, does not behave differently from parental, wild-type BR.

Despite the unaltered growth properties and the fact that use of GFP-expressing recombinant CPXV.BR is very useful for some applications, we further sought to develop the infectious clone in a fashion that would render the geno- and phenotype of viruses reconstituted from the infectious clone absolutely identical to that of parental CPXV.BR. We previously reported that loss of vector sequences is made possible by the occurrence of two antiparallel recombination events mediated by a sequence duplication within mini-F vector sequences [10]. Such recombination in the case of transfection of pBR indeed led to self-excision of the mini-F cassette upon vBR reconstitution, which was shown by the loss of sequences coding for GFP and was confirmed not only by the absence of autofluorescence but also by PCR, RFLP and Southern blotting of virus progeny resulting from pBR transfection.

Genetic identity of the generated CPXV.BAC was proven by RFLP, Southern blot analyses and finally pyrosequencing of the entire cloned genome. Parts of the inverted terminal repeat regions (nt positions 0-2 546 and 232 280-234 825) could not be analyzed due to the high content of A and T rich repeats and the highly repetitive character of the sequences. Remaining sequence differences between the generated BAC and the published sequence available for BR (genbank_accession#NC_003663) were shown to be caused by alterations already present in the parental wild-type CPXV.BR used for cloning or representing errors that occurred following PCR amplification of the TK flanks. Our sequencing results indicate that CPXV.BR isolates differ minimally in their genetic material, an alternative explanation remaining that sequencing errors account for the discrepancies. However, all sequence modifications are located within the less conserved terminal part of the genome, where higher mutation rates due to host-pathogen co-evolutionary events are expected and frequently seen [21]. The fact that congruence was observed regarding the phenotype in vitro was shown by observing plaque sizes and growth kinetics suggests that both generated BAC clones after reconstitution in cultured cells in fact behave in a fashion that is practically identical to that of wild-type virus and present a number of advantages. pBRf, expressing GFP under a poxvirus promoter, produces labeled virus after reconstitution and mutants can easily be tracked by fluorescence microscopy, whilst pBR results in a virus that does not contain any foreign sequences. Therefore, if one generates mutant genomes based on pBR, only the desired mutation will be present and otherwise represent a wild-type background, which will exclude unintended side effects by marker sequences especially in in vivo experiments.

In summary, both BAC clones presented here enable the use of fast and easy bacterial mutagenesis methods for the generation of cowpox virus mutants. In our initial mutagenesis attempts of pBR and pBRf, we observed genomic stability as was shown for the other available orthopoxvirus full-length DNA clones. Therefore, the CPXV.BR BAC represents a toolbox that will simplify pathogenesis studies of a recently emerging virus not only relevant to several species of animals but also exhibiting zoonotic potential. Other members of the family *Poxviridae *have already proven to show excellent performance as vectors for vaccination or gene therapy purposes, applications that are greatly simplified by the use of cloned viral genomes such as the CPXV BAC generated here.

## Competing interests

The authors declare that they have no competing interests.

## Authors' contributions

SJR carried out the molecular cloning and virological studies. SF participated in construction of the plasmids and bacmids. DH, MB and BKT did the nucleotide sequencing and analysis. SJR, MB, BKT and NO designed the experiments. SJR, DH and NO wrote the manuscript. All authors read and approved the final manuscript.

## References

[B1] Johnston JB McFadden G Poxvirus immunomodulatory strategies: current perspectives J Virol 2003 77 6093 6100 10.1128/JVI.77.11.6093-6100.2003 12743266 PMC155018

[B2] Seet BT Johnston JB Brunetti CR Barrett JW Everett H Cameron C Sypula J Nazarian SH Lucas A McFadden G Poxviruses and immune evasion Annu Rev Immunol 2003 21 377 423 10.1146/annurev.immunol.21.120601.141049 12543935

[B3] Shchelkunov SN Safronov PF Totmenin AV Petrov NA Ryazankina OI Gutorov VV Kotwal GJ The genomic sequence analysis of the left and right species-specific terminal region of a cowpox virus strain reveals unique sequences and a cluster of intact ORFs for immunomodulatory and host range proteins Virology 1998 243 432 460 10.1006/viro.1998.9039 9568042

[B4] Shizuya H Birren B Kim UJ Mancino V Slepak T Tachiiri Y Simon M Cloning and stable maintenance of 300-kilobase-pair fragments of human DNA in Escherichia coli using an F-factor-based vector Proc Natl Acad Sci USA 1992 89 8794 8797 10.1073/pnas.89.18.8794 1528894 PMC50007

[B5] Messerle M Crnkovic I Hammerschmidt W Ziegler H Koszinowski UH Cloning and mutagenesis of a herpesvirus genome as an infectious bacterial artificial chromosome Proc Natl Acad Sci USA 1997 94 14759 14763 10.1073/pnas.94.26.14759 9405686 PMC25110

[B6] Brune W Messerle M Koszinowski UH Forward with BACs: new tools for herpesvirus genomics Trends Genet 2000 16 254 259 10.1016/S0168-9525(00)02015-1 10827452

[B7] Domi A Moss B Cloning the vaccinia virus genome as a bacterial artificial chromosome in Escherichia coli and recovery of infectious virus in mammalian cells Proc Natl Acad Sci USA 2002 99 12415 12420 10.1073/pnas.192420599 12196634 PMC129459

[B8] Domi A Moss B Engineering of a vaccinia virus bacterial artificial chromosome in Escherichia coli by bacteriophage lambda-based recombination Nat Methods 2005 2 95 97 10.1038/nmeth734 15782205

[B9] Cottingham MG Andersen RF Spencer AJ Saurya S Furze J Hill AV Gilbert SC Recombination-mediated genetic engineering of a bacterial artificial chromosome clone of modified vaccinia virus Ankara (MVA) PLoS One 2008 3 e1638 10.1371/journal.pone.0001638 18286194 PMC2242847

[B10] Tischer BK Kaufer BB Sommer M Wussow F Arvin AM Osterrieder N A self-excisable infectious bacterial artificial chromosome clone of varicella-zoster virus allows analysis of the essential tegument protein encoded by ORF9 J Virol 2007 81 13200 13208 10.1128/JVI.01148-07 17913822 PMC2169085

[B11] Morgan RW Cantello JL McDermott CH Transfection of chicken embryo fibroblasts with Marek's disease virus DNA Avian Dis 1990 34 345 351 10.2307/1591417 2164390

[B12] Tischer BK Smith G Osterrieder N En passant Mutagenesis - A two step markerless Red recombination system 2010 Humana Press Inc., Tatowa 10.1007/978-1-60761-652-8_30 20677001

[B13] Tischer BK von Einem J Kaufer B Osterrieder N Two-step red-mediated recombination for versatile high-efficiency markerless DNA manipulation in Escherichia coli Biotechniques 2006 40 191 197 10.2144/000112096 16526409

[B14] Russell JSaDW Molecular Cloning 2001 3 New York: Cold Spring Harbor Laboratory Press

[B15] Wiley G Macmil S Qu C Wang P Xing Y White D Li J White JD Domingo A Roe BA Methods for generating shotgun and mixed shotgun/paired-end libraries for the 454 DNA sequencer Curr Protoc Hum Genet 2009 61 18.1.1 18.1.21 10.1002/0471142905.hg1801s61 19360698

[B16] Pennington TH Isatin-beta-thiosemicarbazone causes premature cessation of vaccinia virus-induced late post-replicative polypeptide synthesis J Gen Virol 1977 35 567 571 10.1099/0022-1317-35-3-567 881619

[B17] Woodson B Joklik WK The inhibition of vaccinia virus multiplication by isatin-beta-thiosemicarbazone Proc Natl Acad Sci USA 1965 54 946 953 10.1073/pnas.54.3.946 5217472 PMC219769

[B18] Wittek R Organization and expression of the poxvirus genome Experientia 1982 38 285 297 10.1007/BF01949349 6281055

[B19] Meyer H Sutter G Mayr A Mapping of deletions in the genome of the highly attenuated vaccinia virus MVA and their influence on virulence J Gen Virol 1991 72 Pt 5 1031 1038 10.1099/0022-1317-72-5-1031 2033387

[B20] Weir JP Bajszar G Moss B Mapping of the vaccinia virus thymidine kinase gene by marker rescue and by cell-free translation of selected mRNA Proc Natl Acad Sci USA 1982 79 1210 1214 10.1073/pnas.79.4.1210 6280173 PMC345931

[B21] Committee on the Assessment of Future Scientific Needs for Live Variola Virus IoM Live Variola Virus: Considerations for Continuing Research 2009 1 National Academies Press 25101435

